# The Impact of a Life Story Intervention on Depressive Symptoms of Long-Term Care Residents with Dementia

**DOI:** 10.1093/geroni/igaf122.3666

**Published:** 2025-12-31

**Authors:** Lacey DiFranco, Silvia Orsulic-Jeras, Sara Powers, Farida Ejaz, Lisbeth Sanders

**Affiliations:** Benjamin Rose Institute on Aging, Cleveland, Ohio, United States; Benjamin Rose Institute on Aging, Cleveland, Ohio, United States; Benjamin Rose Institute on Aging, Cleveland, Ohio, United States; Benjamin Rose Institute on Aging, Cleveland, Ohio, United States; LifeBio, Marysville, Ohio, United States

## Abstract

Reminiscence therapies have been shown to positively impact cognition, mood, and other behavioral and psychological symptoms in persons living with dementia (PLWD). LifeBio, a life story intervention, has shown promise in reducing depressive symptoms in nursing home residents with dementia. To address the paucity of scalable life story interventions, a technological adaptation of LifeBio was conducted. LifeBio Memory™, utilizes text-to-speech technology to record resident interviews; machine learning and artificial intelligence enabled production of life story products, such story books, one-page resident bios, and care plans for staff to connect with residents during social or care-based interactions. This paper describes two quasi-experimental, pre–post studies conducted three years apart, testing each version of LifeBio: the first (n = 238) utilized the original, rudimentary protocol and the second (n = 107), used the tablet-based LifeBio Memory™. Both studies demonstrated reductions in depressive symptomology. Study 1, conducted in 16 Ohio nursing homes, showed a significant decrease from baseline (M = 5.59, SD = 5.10) to follow-up (M = 4.71, SD = 5.46); t(168) = 2.46, p = 0.015, using the Patient Health Questionnaire (PHQ-8). Study 2, conducted in 11 distinct Ohio nursing homes, also showed a significant decrease from baseline (M = 11.23, SD 8.56) to follow-up (M = 9.87, SD = 8.04); t(106) = 1.99, p = 0.024 using the Center for Epidemiologic Studies Depression Scale (CES-D). Future research plans to evaluate LifeBio Memory™ in randomized trials, identify mechanisms of reducing depression, and assess cost-effectiveness to inform integration into routine dementia care.

